# Differential preservation of endogenous human and microbial DNA in dental calculus and dentin

**DOI:** 10.1038/s41598-018-28091-9

**Published:** 2018-06-29

**Authors:** Allison E. Mann, Susanna Sabin, Kirsten Ziesemer, Åshild J. Vågene, Hannes Schroeder, Andrew T. Ozga, Krithivasan Sankaranarayanan, Courtney A. Hofman, James A. Fellows Yates, Domingo C. Salazar-García, Bruno Frohlich, Mark Aldenderfer, Menno Hoogland, Christopher Read, George R. Milner, Anne C. Stone, Cecil M. Lewis, Johannes Krause, Corinne Hofman, Kirsten I. Bos, Christina Warinner

**Affiliations:** 1 Department of Archaeogenetics, Max Planck Institute for the Science of Human History, Jena, 07745 Germany; 2 Department of Anthropology, University of Oklahoma, Norman, Oklahoma 73019 USA; 3 Laboratories of Molecular Anthropology and Microbiome Research, University of Oklahoma, Norman, Oklahoma 73019 USA; 4 Faculty of Archaeology, Leiden University, Leiden, 2333 CC The Netherlands; 5 Natural History Museum of Denmark, University of Copenhagen, Copenhagen, 1350 Denmark; 6 Center for Evolution and Medicine, Arizona State University, Tempe, Arizona 85287 USA; 7 Department of Microbiology and Plant Biology, University of Oklahoma, Norman, Oklahoma 73019 USA; 8 Grupo de Investigación en Prehistoria IT-622-13 (UPV-EHU)/IKERBASQUE-Basque Foundation for Science, Vitoria, Spain; 9 Department of Anthropology, Dartmouth College, Hanover, New Hampshire 03755 USA; 10 Department of Anthropology, Smithsonian Institution, Washington DC, 20560 USA; 11 School of Social Sciences, Humanities, and Arts, University of California Merced, Merced, California 95343 USA; 12 Institute of Technology Sligo, Sligo, F91 YW50 Ireland; 13 Department of Anthropology, Penn State University, University Park, Pennsylvania 16802 USA; 14 School of Human Evolution and Social Change, Arizona State University, Tempe, Arizona 85287 USA; 15 Institute of Human Origins, Arizona State University, Tempe, Arizona 85287 USA

## Abstract

Dental calculus (calcified dental plaque) is prevalent in archaeological skeletal collections and is a rich source of oral microbiome and host-derived ancient biomolecules. Recently, it has been proposed that dental calculus may provide a more robust environment for DNA preservation than other skeletal remains, but this has not been systematically tested. In this study, shotgun-sequenced data from paired dental calculus and dentin samples from 48 globally distributed individuals are compared using a metagenomic approach. Overall, we find DNA from dental calculus is consistently more abundant and less contaminated than DNA from dentin. The majority of DNA in dental calculus is microbial and originates from the oral microbiome; however, a small but consistent proportion of DNA (mean 0.08 ± 0.08%, range 0.007–0.47%) derives from the host genome. Host DNA content within dentin is variable (mean 13.70 ± 18.62%, range 0.003–70.14%), and for a subset of dentin samples (15.21%), oral bacteria contribute > 20% of total DNA. Human DNA in dental calculus is highly fragmented, and is consistently shorter than both microbial DNA in dental calculus and human DNA in paired dentin samples. Finally, we find that microbial DNA fragmentation patterns are associated with guanine-cytosine (GC) content, but not aspects of cellular structure.

## Introduction

Dental calculus is a mineralized form of dental plaque^1^, a sequentially generated microbial biofilm^2^ that entraps microbial, dietary, host, and ambient debris during spontaneous calcification events^3^. Unlike body mucosal surfaces that have continual cell turnover, teeth do not remodel. Consequently, they are relatively stable environments for bacterial colonization during biofilm development^4^, making the formation of dental calculus difficult to prevent without mechanical removal. As a result, dental calculus is prevalent in the archaeological record, and due to its excellent morphological preservation, it has long been an attractive target for microscopic analysis^5–11^. More recently, dental calculus has been explored as a source of ancient DNA (aDNA) and has been shown to retain an excellent record of the human oral microbiome^12–15^. It may provide insights on ancient diet through isotopic analysis^16^, and it serves as an alternative source of endogenous host DNA^17^.

Retrieving viable aDNA from archaeological sources, whether from skeletal tissues (bone and dentin) or from secondary substrates (dental calculus and paleofeces), is challenged by post-mortem decomposition, where temporal and environmental factors compromise the molecular stability of DNA. These degradative processes include oxidative and hydrolytic damage to individual bases, hydrolytic lesions on the sugar-phosphate backbone, DNA fragmentation due to nuclease activity, and general degradation by microorganisms involved in the decomposition process^18,19^. As a result, there is a reduction in the amount of recoverable DNA, and aDNA that is retrieved is characterized by predictable forms of damage, including extreme fragmentation, depurination, and high-levels of terminal cytosine deamination^19–21^. In addition to damage, ancient samples can acquire exogenous contamination that may obscure any remaining endogenous signal. Susceptibility to contamination and molecular degradation appears to be tissue specific, with petrous bone and tooth cementum generally exhibiting the highest proportions of endogenous human DNA among archaeological skeletal and soft tissues^22–25^. Recent studies have suggested that dental calculus may be more resistant to environmental contamination than other sources of aDNA, and higher overall DNA yields have been reported from dental calculus than from any other archaeological source^14,17^. While these attributes of dental calculus are compelling, they have been reported from a small number of samples that have been insufficiently explored for additional variables such as temporal age, depositional context, or geographic location^14,17,26^. Therefore, the prospect of ancient dental calculus as a dependable source of well-preserved, endogenous aDNA (both host and oral microbiome derived) has yet to be rigorously and systematically tested.

**Figure 1:**
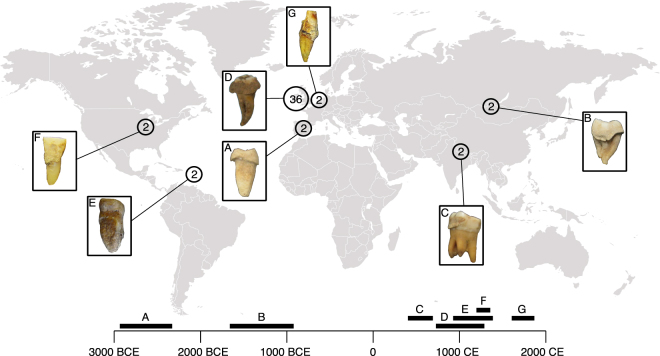
Geographic locations and temporal periods of archaeological teeth included in this study. **(a)** Camino del Molino, Iberia; **(b)** Arbulag Soum, Khövsgöl, Mongolia; **(c)** Samdzong, Nepal; **(d)** Kilteasheen, Ireland; **(e)** Anse à la Gourde, Guadeloupe; **(f)** Norris Farms, Illinois, USA; **(g)** Middenbeemster, the Netherlands. For each site, representative teeth with dental calculus deposits are shown in boxes. The number of teeth (dentin-calculus pairs) analyzed per site is provided within the indicated circles, and corresponding letters on the timeline indicate the time period represented by each site.

In the present study, metagenomic sequence data from 48 paired dentin and dental calculus samples are compared to determine whether endogenous DNA exhibits a different degree of preservation in dental calculus than in dentin from the same individual. Individuals included in this study represent seven archaeological sites spanning diverse geographic, environmental, and temporal ranges. Thirty-six paired samples were selected from a single medieval cemetery in Kilteasheen, Ireland (here referred to as the regional sample set) in order to examine intra-site variation in preservation. Additionally, 12 paired samples from six different archaeological sites on three continents spanning a broad temporal range were selected to control for environment, burial context, time period, and individual dynamics that may impact preservation quality (here referred to as the global sample set) (Fig. 1; Supplementary Table S1). We compare DNA preservation within archaeological dental calculus and dentin from the same tooth, with a specific focus on four main measures: (1) DNA abundance, (2) microbial community composition and contamination, (3) human DNA content, and (4) DNA fragmentation and damage patterns. Our findings confirm that calculus yields more total DNA when compared to dentin, with a low, albeit consistent, proportion of endogenous human DNA. Microbial profiles of dental calculus suggest that it retains a robust signal of the human oral microbiome and is relatively resistant to exogenous contamination, while dentin is typically dominated by environmental microbial sources. A subset of dentin, however, contains DNA from oral bacteria (possibly deriving from postmortem colonization during decomposition processes), indicating that dentin may serve as an alternative source for DNA from individual oral microbes in some cases. With respect to DNA degradation, we find that DNA fragmentation patterns within dental calculus are associated with the genomic source of the DNA (human vs. microbial) but not with cellular structure (e.g., microbial cell wall type or presence of a surface-layer). Additionally, human DNA is consistently shorter in dental calculus than in paired dentin samples, which may reflect differences in the manner by which human DNA is incorporated into each matrix. Finally, we observe a systematic loss of short AT-rich DNA fragments that is particularly marked in bacteria with low to medium GC content genomes.

## Results

### DNA abundance

**Figure 2:**
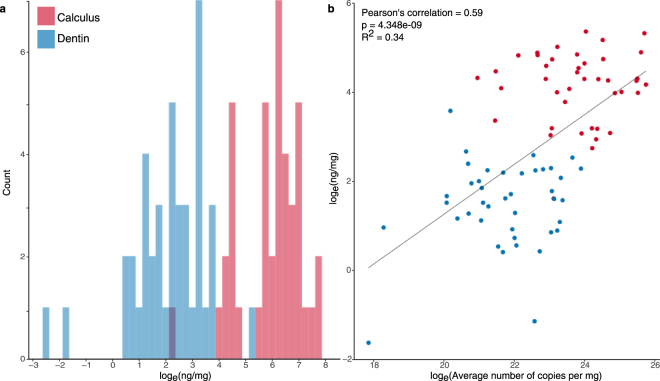
Total DNA content of dental calculus is higher than dentin as measured by both fluorescence and quantitative PCR (qPCR) techniques. (**a**) Normalized DNA yield (log transformed nanograms DNA per milligram starting material) of DNA extracts obtained from dental calculus and dentin as measured by a Qubit fluorometer using a High Sensitivity Assay (*p* = 3.911e-11, Wilcoxon signed-rank paired test). (**b**) Linear correlation between normalized DNA yield (log(ng/mg)) and normalized DNA yield as measured by qPCR after library preparation. The average number of copies per milligram was calculated from the mean of four technical replicates, and these values were compared between dentin and calculus (*p* = 6.941e-07, Wilcoxon signed-rank paired test). In both quantification metrics dental calculus has a higher DNA yield than dentin.

The total amount of DNA recovered from dental calculus is substantially higher than from dentin as measured by both fluorometric quantitation (Qubit) (Fig. 2a) and quantitative PCR (qPCR) (Fig. 2b). Immediately following extraction, DNA yields from dental calculus as measured by fluorometry ranged from 4.9 ng/mg to 214.4 ng/mg (median 72.1 ng/mg), while dentin samples from the same individuals yielded 0.2 ng/mg to 35.7 ng/mg (median 4.8 ng/mg). These measurements are moderately correlated (Pearson’s coefficient: 0.59; 95% CI: 0.43, 0.72) to the average DNA copy number estimated after library construction using qPCR. This fit accounts for 34% of the total variance using a linear regression model (Fig. 2b). Differences between the two methods may be artifacts of DNA library construction, during which inefficient ligation of universal adapters to the DNA fragments and several silica-based purification steps contribute to substantial, but stochastic, DNA loss^27^. While differences in DNA abundance are not as stark in the qPCR results, the overall amount of DNA recovered from dental calculus is significantly higher than from dentin using both metrics (fluorometry, *p* = 7.822e-11, Wilcoxon signed-rank paired test; qPCR, *p* = 6.941e-07, Wilcoxon signed-rank paired test).

### Microbial community composition and contamination

**Figure 3:**
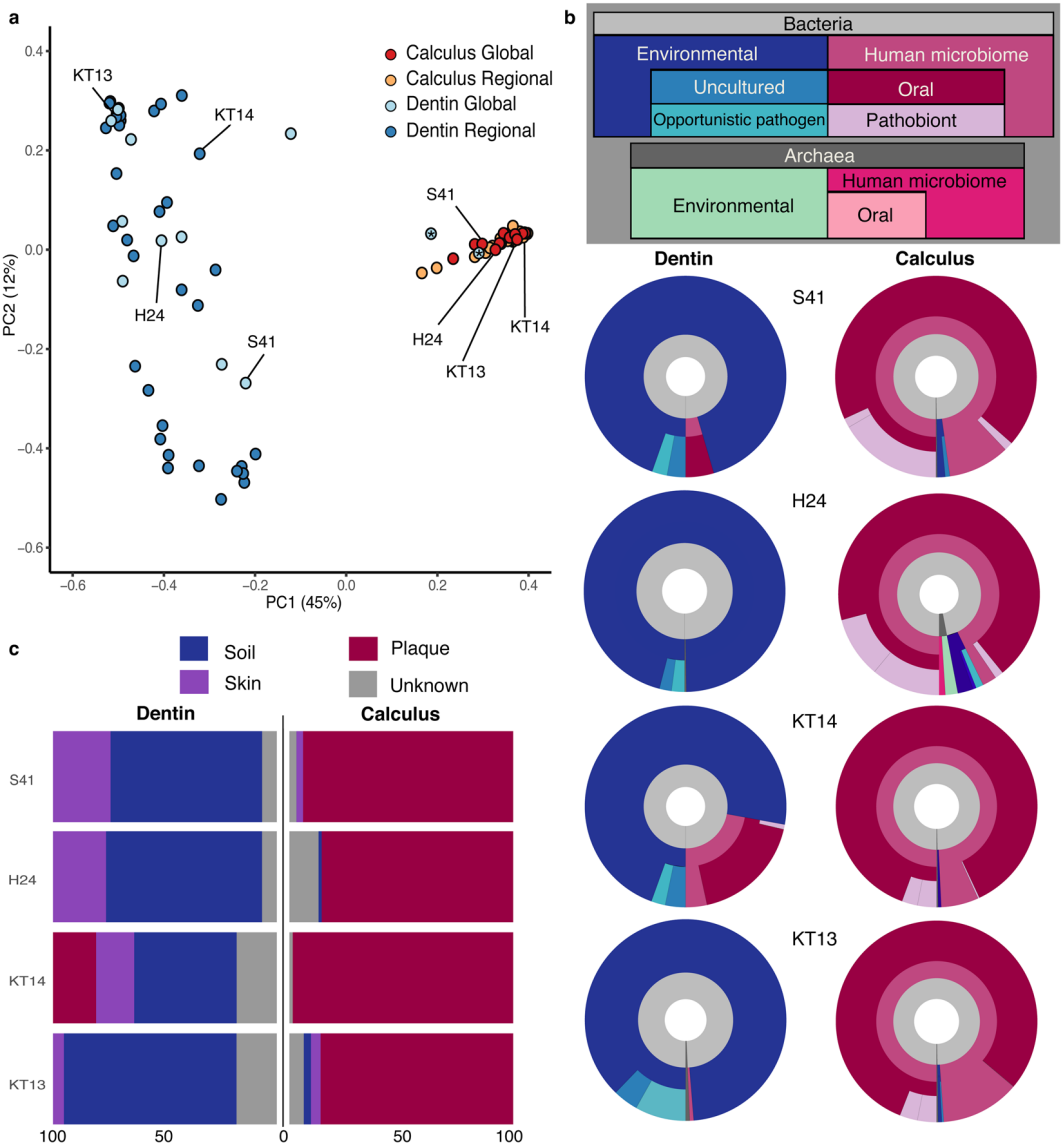
Microbial communities represented in archaeological dental calculus and dentin are distinct. (**a**) Principal Coordinates Analysis (PCoA) of Bray-Curtis distances of all bacterial and archaeal species-level assignments from dental calculus and dentin. Color indicates material type (dental calculus or dentin) and membership in global or regional dataset. Microbial taxonomy was assigned using MALT with the full NCBI nucleotide database. Dentin samples marked with an asterisk belong to individuals NF47 and NF217 in the global dataset, and may represent teeth with carious lesions (Supplementary Figure S2). (**b**) A subset of four sample pairs from the global (S41, H24) and regional (KT14, KT13) sample sets were selected to further demonstrate differences in dental calculus and dentin microbial communities. Species represented in the MALT results were sorted into a layered classification scheme (see legend in gray box), and the proportions of reads assigned to each taxon were used to generate Krona plots. (**c**) Stacked bar plots of Bayesian SourceTracker results for the four selected pairs display estimated proportions of source contribution at the genus level, using modern plaque, skin, and soil datasets as model sources. Both approaches show overwhelming abundance of environmental bacteria within dentin samples, while most microbial DNA within the paired calculus samples are associated with, and most likely derive from, the human oral microbiome. The robustness of the human microbiome signal to the environmental signal is driving the separation we see in the PCoA.

Overall, archaeological dental calculus and dentin contain microbial DNA from highly distinct communities. To explore these differences, shotgun-sequenced reads from the 48 dentin and dental calculus pairs were taxonomically binned for microbial identification using MALT^28^, and the resulting assignments were visualized using MEGAN^29^ (Supplementary Tables S2 and S3). A species-level PCoA plot based on a Bray-Curtis dissimilarity matrix demonstrates that dental calculus samples form a relatively tight and cohesive group that is distinct from the more diffuse distribution of microbial communities identified within dentin (Fig. 3a). Alpha diversity is significantly higher for dentin samples as compared to dental calculus (Mann-Whitney-U, *p* = 8.55E-15), consistent with a signal of external contamination (Supplementary Figure S1). Differences in microbial community structure and composition between dentin, calculus, and blanks was further explored using linear discriminant analysis^30^. Dentin and blank samples are primarily discriminated by taxa that are environmentally abundant, while calculus samples are discriminated by typically oral taxa (see Supplementary Methods; Supplementary Table S4). Importantly, microbial communities from each material (dental calculus or dentin) are less similar to their paired sample than they are to samples of the same material. This pattern is consistent with expectations that the microbial taxa within dental calculus represent a relatively well preserved biological community derived from dental plaque, while dentin – being sterile in life – is expected to harbor a microbial community primarily composed of exogenous contaminant bacteria acquired through stochastic postmortem processes.

Two additional approaches were used to characterize further the nature and inferred sources of the microbial communities present within sample-types. First, microbial species-level identifications were categorized according to a nested scheme reflecting organism membership in one or more of the following source categories: environmental, uncultured environmental, human microbiome, human oral, pathobiont, and opportunistic pathogen (Fig. 3b; Supplementary Table S5), which were then visualized using the Krona Excel template^31^. Category membership was determined by species presence or absence in the Human Oral Microbiome Database^32^ as well as source and habitat descriptions in the twenty most recent articles in PubMed^33^ using the species name as the search keyword. Using this analysis, stark differences are observed in the inferred microbial source contributions to archaeological dental calculus and dentin, whereby dental calculus is strongly dominated by human-associated – and especially oral-associated – taxa, while dentin is primarily composed of environmental taxa (Supplementary Table S6).

As a separate confirmation method, SourceTracker, a Bayesian source-prediction tool^34^, was used to estimate proportions of source similarity (Supplementary Table S7) in dental calculus and dentin based on a set of modern reference microbial communities sequenced from human dental plaque^35,36^, human hand swabs^37^, and top soil samples from Oklahoma and Alaska^38^ (Fig. 3c). The human hand swab reference community was included in this analysis to detect microbial contaminants introduced through handling during excavation or storage. In agreement with the other methods presented here, archaeological dental calculus is estimated to be composed primarily of dental plaque-associated taxa, while dentin is dominated by genera associated with human skin and environmental sources (Supplementary Table S8). The highest predicted contributions of skin and soil to dental calculus samples are 7.9% and 14.0%, respectively, while the same predicted contributions for dentin are 33.5% and 89.0%, respectively. Together, these analyses suggest dental calculus is relatively robust to environmental contamination when compared to dentin.

Although most dentin samples are strongly dominated by environmental taxa, two dentin samples from the site of Norris Farms – NF47 and NF217 – cluster with dental calculus samples in the PCoA (Fig. 3a) and are estimated via SourceTracker analysis^34^ to contain microbial DNA that is 56.2% and 78.4% derived from dental plaque, respectively (Supplementary Figure S2). Unlike other teeth in this study, the Norris Farms teeth were obtained in a fragmented state. Lacking the full intact teeth, the presence of carious lesions could not be ruled out. In addition, because caries are not associated with the presence, absence, or even abundance of specific organisms, we were not able to determine whether a carious lesion was present using metagenomic data alone^39,40^. For this reason, the Norris Farms samples were excluded from further downstream analyses.

In samples for which the tooth was intact, tooth dentin is generally strongly dominated by environmental taxa; however, a subset of dentin samples exhibit a slight signal of the human oral microbiome, ranging from 0.0% to 40.0%, with 7 of 46 dentin samples having a predicted oral source contribution of 20.0% or more by SourceTracker analysis (Supplementary Figure S3). A PCoA and SourceTracker analysis including blanks confirms this oral signal is not due to laboratory-based contamination (Supplementary Figure S4). Based on our classification scheme visualized by Krona plots, microbial DNA derived from human microbiome sources constituted a median of 98% (ranging from 90.1% to 99.3%) of the total microbial contribution in the calculus samples, whereas, for dentin, human microbiome DNA constitutes a median of 3.7% (ranging from 0.2% to 61%; Supplementary Table S6).

### Human DNA content

**Figure 4:**
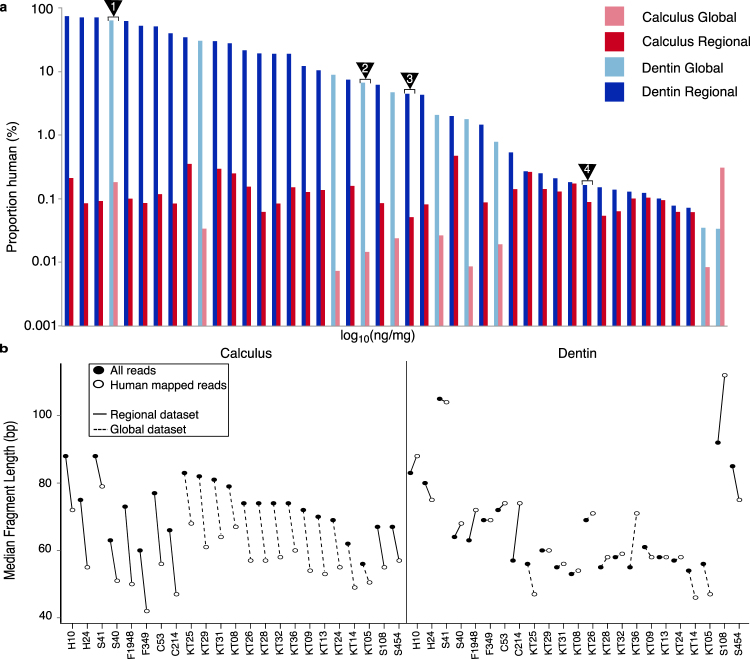
Human DNA in dental calculus shows consistent patterns of low relative abundance and high fragmentation. (**a**) Relative percentage of human DNA in all paired calculus and dentin samples calculated from de-duplicated reads mapped to the hg19 human reference genome using BWA. While the majority of dentin samples have an overall higher percentage of human DNA, this value varies substantially by sample. Calculus is comparatively consistent between samples albeit on average lower than their paired dentin sample. Sample pairs corresponding to those in Fig. 3 are indicated by numbered triangles: (1) S41, (2) H24, (3) KT14, (4) KT13. (**b**) Median fragment length of merged reads mapping to the human genome compared to all merged reads in both dental calculus and dentin. Human assigned reads in dental calculus are shorter than expected independent of age, laboratory processing protocol, or sample preservation. Human mapped reads in dental calculus and dentin were further verified for authenticity using strict mapping parameters (Supplementary Figure S5, Supplementary Table S9).

Although typically higher than in dental calculus, the proportion of human endogenous DNA in dentin varies substantially, ranging more than 3 orders of magnitude in this study, from 0.034% to 70.1% of all reads (Fig. 4a). By contrast, the proportion of human DNA in dental calculus is relatively low, but consistent across all samples, differing by less than 2 orders of magnitude, from 0.007% to 0.47%. To verify these reads as authentic host DNA and to mitigate the possibility that they represent spurious mapping to the human genome or modern contamination, a secondary verification procedure was performed. Here, only those reads that (1) met stringent mapping criteria when mapped to the hg19 human reference genome (see Supplementary Methods), (2) were assigned to the *Homo sapiens* node in lowest common ancestor assignment by MALT, and (3) displayed typical ancient DNA damage profiles^41^ were included in a high confidence human dataset for further inspection.

The number of human-assigned reads following strict mapping decreased across all samples but was more severe among samples from the Kilteasheen (regional) dataset. As the strict mapping parameters allow only one mismatch per 50 base pairs, this comparatively high loss of reads in the regional dataset likely results from the fact that these samples were sequenced using a single-end, 75 cycle sequencing strategy, rather than the paired-end 200 cycle sequencing strategy employed for the global sample set. Of all originally designated human reads in the regional dataset, between 20.7% to 60.9% of the dental calculus reads and 0.6% to 67.1% of dentin reads pass the initial strict mapping step. Within the global dataset, between 60.1% to 88.3% of dental calculus and 74.0% to 97.9% of dentin reads pass.

Reads passing strict mapping were next analyzed with MALT using the full NCBI nucleotide database as a reference to ensure proper assignment to *Homo sapiens* rather than to other organisms. Among all dental calculus samples, the proportion of reads uniquely assigned to the *Homo sapiens* node ranges from 79.6% to 90.3%. Within all dentin samples the proportion of reads assigned to *Homo sapiens* ranges from 81.1% to 92.7%.

Finally, to establish that these reads are consistent with patterns expected of authentic ancient host DNA and not modern contamination, rates of terminal cytosine deamination were evaluated using mapDamage 2.0^41^. While damage rates declined (median 3% in dentin and 4% in calculus) after these verification steps (Wilcoxon sign rank test, dentin *p* = 1.36E-15, calculus *p* = 6.74E-15; See Supplementary Table S9), all samples except one dentin (KT05, Supplementary Figure S5d) present damage patterns consistent with authentic aDNA both pre- and post-verification. This contrasts with contaminant human DNA observed in the extraction and library blanks, which showed no evidence of DNA damage, and thus is consistent with modern human contamination (Supplementary Figure S5; Supplementary Table S10). Because the human DNA sequences present in the blanks lack damage, they are therefore unlikely to be the source of the damaged human reads in the dentin and calculus samples. Taken together, these analyses suggest that although a small subset of reads may be erroneously assigned to the human genome, most reads are likely correctly assigned, and these reads appear to be authentic ancient human DNA and not contamination.

Next, DNA fragmentation and damage patterns of human reads in paired dentin and dental calculus samples were compared for a subset of the samples for which paired-end DNA sequence data was generated (Supplementary Table S11), which includes the entire global sample set (n = 10 individuals) and a representative subset of the regional sample set (n = 13 individuals). Overall, the median fragment lengths of total DNA recovered from both dental calculus (56–88 bp, mean 72.8 bp) and dentin (53–105 bp, mean 66.0 bp) are short and fall within a size range expected for archaeological samples (Supplementary Table S1). For each dental calculus sample, the median fragment length of human reads was found to be 15.5 ± 4.2 bp shorter than the overall median fragment length of DNA in each sample, which is primarily microbial in origin (Fig. 4b). Dentin samples, however, show no pattern with respect to human DNA fragment length compared to overall DNA fragment length. Because some dentin samples have a high endogenous human content, a separate analysis comparing the fragment length profile of human mapped reads and all non-human reads was performed (Supplementary Figure S6). In calculus, there is a greater average magnitude of difference between the mean fragment length of human and non-human reads (calculus: 0.50 ± 0.15; dentin: 0.20 ± 0.17, Cohen’s d), and the median length of non-human reads is consistently longer than that of human reads. Conversely, the magnitude of difference between human and non-human fragment length profiles in dentin is variable and inconsistent (Supplementary Table S11). This analysis confirms that the observed differences in fragment length profiles between dentin and dental calculus is not the result of high human endogenous content of particular samples. Comparing human DNA in dental calculus and dentine, we find that human DNA within dental calculus is generally more fragmented than human DNA in paired dentin samples, with the median length of calculus-derived fragments being approximately 10.3 bp ± 12.0 shorter than that of dentin-derived fragments (Wilcoxon signed-rank test, *p* = 0.01178); however, this pattern is largely driven by the relatively long human DNA fragment lengths in dentin, and further work is needed to determine if this is an artifact of sample preparation or a true biological pattern.

We next investigated the relative degree of terminal cytosine deamination among human reads in dental calculus and dentin pairs from the same individual (Supplementary Figure S7). Across all samples, the terminal cytosine deamination rate of dentin is significantly higher than dental calculus (*p* = 0.0009, Wilcoxon signed-rank test), yet there is no significant difference in the cytosine deamination in overhangs (δ_d_, *p* = 0.49, Wilcoxon signed-rank test) or in double stranded regions (δ_s_, *p* = 0.07, Wilcoxon signed-rank test). However, the average length of overhangs (λ) is significantly different between all dentin and calculus samples (*p* = 0.04, Wilcoxon signed-rank test) (Supplementary Table S9). These results suggest that while differential damage parameters may not be consistent between paired samples, some patterns may be predicted by sample type.

### Microbial DNA fragmentation and damage patterns

**Figure 5:**
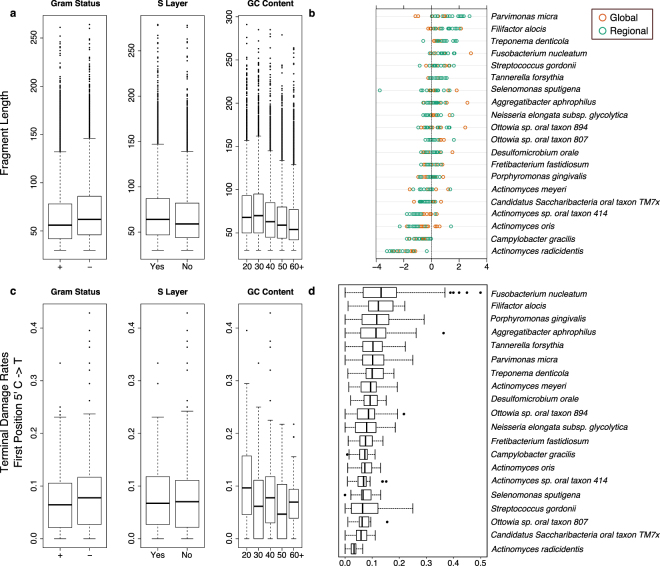
Fragment length and damage rates among bacterial taxa within calculus. (**a)** Fragment length distribution of 50 high frequency species-level bacteria among all dental calculus grouped into three metadata categories: gram status, the presence or absence of a surface layer (S-layer), and the overall genomic GC content of the organism as documented from the reference genome in the NCBI database. Input was normalized to 400 randomly chosen reads per sample per species to mitigate the impact of sample specific read length profiles. No detectable differences in the median are apparent for the presence of an S-layer or gram status. Genomic GC content, however, does impact the overall fragment length profile of reads. **(b)** Deviation of the median fragment length from overall sample median fragment length of a subset of 20 oral bacteria in dental calculus colored by dataset origin. Again, genomic GC content appears to impact the fragment length profiles of individual taxa where high GC content species have shorter overall fragment length profiles. **(c)** Terminal cytosine damage rates (C to T substitution ratio at the first position of the 5′ end of the molecule) among 50 bacterial species in dental calculus grouped by gram status, presence or absence of an S-layer, and overall genomic GC content. No detectable differences in damage rates could be detected in any of the three chosen metadata categories. **(d)** Terminal cytosine damage rates (C to T substitution ratio at the first position of the 5′ end of the molecule) among 20 oral bacteria. While some differences in damage patterns can be detected, it is unclear if this is random or due to some process not examined in this study.

We next investigated fragmentation and terminal cytosine damage patterns for a selection of oral microbes preserved at high abundance within dental calculus in order to determine the impact of cell wall or genomic structure on aDNA preservation (Supplementary Table S12). It has been previously suggested that cell wall composition may influence the preservation of microbial DNA in archaeological dental calculus and dentin^13,42^. Fifty oral bacteria were selected based on their frequency in the dataset and presence in the Human Oral Microbiome Database^32^ and categorized into groups based on whether they are known to be Gram stain positive or Gram stain negative, the presence or absence of a surface layer (S-layer), and overall genomic GC content (Figs 5a and c). Additionally, a subset of 20 of these taxa that were highly abundant in our dataset was analyzed to examine species-level patterns of fragmentation (Fig. 5b) and DNA damage (Fig. 5d).

We found no indication of a relationship between terminal cytosine damage and microbial genomic source (Fig. 5c,d, Supplementary Table S13) nor a relationship between fragmentation and cell wall structure. However, we do see a small decline in average DNA fragment length in taxa with higher genomic GC content (Fig. 5a). This pattern is also reflected among the 20-species subset of oral bacteria chosen for closer analysis, and reads assigned to *Actinomyces radicidentis*, the species with the highest GC content, have the largest displacement from the median fragment length of all selected taxa (Fig. 5b).

### GC content shifts

**Figure 6:**
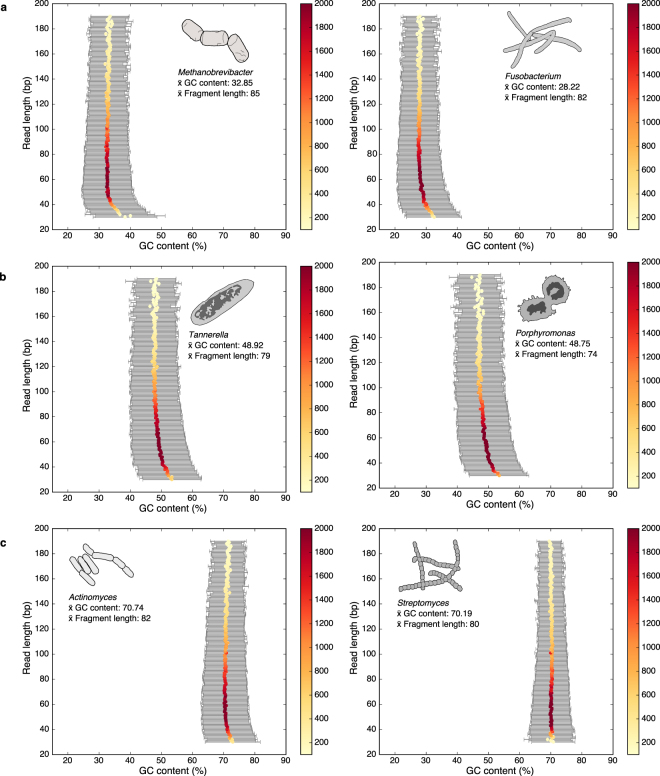
Relationship of GC content to fragment length in five prevalent oral genera and one soil genus (*Streptomyces*). (**a)** Two low expected GC content genera, *Methanobrevibacter* and *Fusobacterium* binned by read lengths wherein each dot represents the mean GC content for that bin. **(b)** Two moderate expected GC content general, *Tannerella* and *Porphyromonas* binned by read lengths wherein each dot represents the mean GC content for that bin. **(c)** One high expected GC content oral genus (*Actinomyces*) and one high expected GC content soil genus (*Streptomyces*) binned by read lengths wherein each dot represents the mean GC content for that bin. Heat bar indicated the number of reads representing each length bin. In all cases except for *Streptomyces* only reads from dental calculus samples were included. Reads for *Streptomyces* were extracted from dentin samples to contrast GC shifts in a potentially modern soil contaminate with typical members of the human oral microbiome. In all cases except for *Streptomyces* the GC content of shorter reads is skewed higher than the overall mean value. This effect greater in low to moderate expected GC content genera than those with higher GC content (Supplementary Figure S8).

Finally, the relationship between fragment length and mean GC content was examined for five prevalent oral genera in dental calculus samples and a single prevalent soil genus in dentin samples to evaluate further the influence of genomic structure on microbial DNA survival. Genera were chosen to maximize the range of GC content with two genera each representing low, medium, and high-expected genomic GC content (Fig. 6). Among all published genomes available in the NCBI genome database^33^, members of the genus *Methanobrevibacter* range in genomic GC content from 24.2% in *M*. *wolinii* to 32.6% in *M*. *ruminantium*. The common oral methanogen *M*. *oralis* is expected to have a GC content of 27.9%. Members of *Fusobacterium* also have low GC content, ranging from 26.0% (*F*. *perfoetens*) to 35.1% (*F*. *necrophorum*). Among the medium GC content genera, the GC content of *Tannerella* ranges from 37.7% (*T*. *sp*. CAG:118) to 56.5% (*T*. *sp*. oral taxon HOT-286), and *Porphyromonas* range from 42.7% (*P*. *gingivicanis*) to 56.3% (*P*. *bennonis*). Finally, for high GC content genera, *Actinomyces* ranges from 49.6% (*A*. *coleocanis*) to 73.1% (*A*. *dentalis*), and *Streptomyces* ranges from 56.4% (*S*. *sp*. WAC00263) to 71.1% (*S*. *sp*. NBRC110027). In comparing reads assigned to these genera in our samples, we detect an increase in GC content at shorter read lengths among all chosen genera except for *Streptomyces*, where no shift was observed. Importantly, this shift is greater for genera with moderate and low expected GC content. For example, in *Methanobrevibacter* and *Fusobacterium* the length at which the mean GC content begins to substantially shift (≥1 z score) is 39 bp and 48 bp, respectively (Supplementary Figure S8). This shift occurs at longer lengths for *Tannerella* and *Porphyromonas* at 59 bp and 58 bp, respectively, while *Actinomyces* does not present a substantial shift until 35 bp. No shift is observed in *Streptomyces*, although this may occur in short fragments that are below the length-filtering threshold (30 bp) used for this dataset.

## Discussion

### Dental calculus is a richer source of genetic material than dentin

In agreement with the findings of previous studies^14,17^, overall DNA recovery from ancient dental calculus was found to be substantially higher than from dentin, and this pattern is consistent through time and across preservation contexts. This higher DNA content of archaeological dental calculus compared to dentin likely reflects biological differences between the two substrates in cellular composition and structure during life, as well as decomposition patterns after death.

Dental calculus is formed from dental plaque, a dense microbial biofilm that has been estimated to contain more than 200 million cells per milligram^43^. Approximately 70% of the dry weight of plaque consists of microbial cells^44^, and a large proportion of the biofilm matrix itself is composed of extracellular bacterial DNA, which provides both structural support and protection to its microbial inhabitants^45^. Furthermore, the mineralization process that leads to dental calculus formation involves rapid inter- and intracellular crystal formation by calcium phosphates, including hydroxyapatite, which strongly bind DNA. The result is a dense crystalline structure that is relatively inert and resistant to microbial attack, enzymatic action, and non-acidic chemical alteration^46^. Although the surface of dental calculus remains porous^47^, penetration of substances towards the internal layers of the calculus matrix is restricted^48^, which may account for its high DNA preservation qualities. Calculus is not an entirely closed system, however. It has been shown to disproportionately lose soluble small metabolites over time^49^, suggesting that the mineralized matrix allows some degree of water movement.

In contrast to dental calculus, dental hard tissues are largely acellular, with live cells in mature teeth being limited to a layer of odontoblasts lining the pulp chamber wall, a sparse distribution of entrapped cementocytes within apical cementum, and a layer of cementoblasts around the periodontal ligament^44,50,51^. Most cells within teeth are instead found within the dental pulp and consist of perivascular cells, blood cells, and pulpal blood vessels^44,52^, all of which decompose readily after death through a combination of necrosis and microbial invasion^3^. Thus, while the majority of cells within dental calculus are found within a mineralized structure conducive to preservation, the majority of cells within teeth are not, which may partially explain the large differences in total DNA yield between the two substrates. However, further studies of total DNA yields from freshly extracted teeth and their component tissues are needed to fully understand these differences.

### Dental calculus and dentin harbor distinct microbial communities

Microbial DNA obtained from dental calculus and dentin derives from distinct communities. In agreement with previous studies^3,14^, the microbial community in dental calculus is dominated by human-associated oral taxa, and DNA derived from these organisms greatly exceeds that originating from environmental sources. The consistent preservation of a strong oral microbiome signal in all 48 dental calculus samples in this study suggests that this pattern is typical for archaeological dental calculus. By contrast, microbial DNA within dentin primarily derives from environmental sources. This distinction between the two substrates is preserved across geography, burial environment, and temporal period.

The tight clustering of all calculus samples included in this study, in contrast to the diffuse distribution of dentin samples in the PCoA (Fig. 3a), indicates that the oral microbiome signal is relatively uniform and stable across diverse contexts, as expected for a preserved biological community. In comparison, the diffuse distribution of dentin samples reflects the diverging influences of exogenous microbes from different environmental contexts and the absence of a consistent microbial composition. Despite the presence of DNA belonging to oral microbes in some dentin samples, none of those included in this analysis join the calculus cluster, indicating they are the result of stochastic preservation of particular oral microbes and do not retain a signal of a biological community.

### Dentin is a source of oral microbial DNA

Although most microbial DNA within dentin is environmental in origin, we find that oral bacteria contribute >20% of total DNA in 7 of 46 dentin samples in this study. Notably, the teeth in this study (with the exception of the two excluded Norris Farms teeth) could be confirmed to be free of oral pathology, such as caries, and the dentin collection procedure included either surface cleaning or only the pulp chamber was sampled. Thus, dental infection and incomplete calculus removal are unlikely explanations for the presence of oral microbial DNA we observe in dentin. Our results agree with recently reported data^53^, in which human-associated microbes constituted ~15% of the organisms identified in a metagenomic study of over 100 archaeological, root-derived dentin samples. These findings suggest that members of the oral microbiome may also participate in postmortem dental decomposition, although to a lesser extent than environmental microbes.

The presence of DNA from oral taxa in dentin has important implications for the study of ancient commensal microbes and their evolution. Although prevalent, dental calculus is not always present or preserved in archaeological skeletal collections. Additionally, dental calculus may be absent or found in low abundance in young individuals or for certain populations or time periods due to genetic, dietary, or behavioral differences. If insufficient calculus is available for study, it may be possible to instead access aspects of the oral microbiome through tooth dentin. Although the stochastic processes of postmortem microbial growth would preclude oral microbiome community-level analyses, genetic sequencing of dentin could nevertheless provide access to the genomes of individual oral taxa for analysis.

### Dental calculus is a source of host DNA

Although dentin generally contains a higher proportion of total human DNA than dental calculus, many dental calculus samples in this study have comparable proportions of human DNA to their dentin pair, with one dental calculus sample from the Netherlands (S454) having a higher proportion of DNA assigned to the human genome than its paired dentin sample (Supplementary Table S9). This pattern is largely driven by the high variability of human DNA preservation within dentin.

Except in cases of blood-borne infections that infiltrate the pulp chamber and expose pathogen DNA to the dentin matrix^42^, nearly all DNA in dentin should originate from the host genome at the time of death. However, archaeological teeth typically contain low proportions of human DNA due to postmortem degradation and exogenous microbial growth. By contrast, the relative proportion of human DNA is uniformly low and relatively consistent in dental calculus. When dentin is strongly degraded and the relative proportion of host DNA in dentin is very low (<0.1%), the absolute amount of human DNA within dental calculus can exceed that of dentin. In such cases, the genetic richness of dental calculus appears to compensate for its low relative proportion of human DNA. Although obtaining host DNA from dental calculus using shotgun sequencing is generally inefficient given its low relative abundance, dental calculus has been shown to be a valuable reservoir for recovering host DNA using DNA capture methods^17^.

### Human DNA in dental calculus is highly fragmented

We find human DNA from dental calculus to be consistently shorter than the total DNA from the same sample, and on average shorter than human DNA recovered from the paired dentin sample. As a microbial biofilm, dental calculus is not a human tissue and does not contain viable human cells. The mechanisms by which human DNA is incorporated into dental calculus are not well understood but are presumed to include passive adsorption of human DNA from oral fluids and shed mucosal cells, as well as more active incorporation through host inflammatory responses, including a kind of immune response mediated by neutrophils known as NETosis^14,54^. Neutrophils, the most abundant nucleated cell type in human blood, are an essential cell type of the innate immune system that are recruited during active microbial infection^55^. Particularly important in the pathogenesis of periodontal disease, neutrophils are recruited in high numbers into the gingival crevice to attack dental plaque bacteria^45,56^. Previous research has found that most human proteins recovered from both modern and ancient dental calculus are associated with the innate immune system, and specifically with neutrophils^14^.

If host immune activity is a major contributor of human DNA to dental calculus, the role of neutrophils in this activity may partially explain the higher degree of human DNA fragmentation in dental calculus than in dentin. While neutrophils and other immune cells are capable of phagocytizing individual or small aggregates of microbial cells, large pathogens or those that can thwart phagocytosis by forming biofilms stimulate the formation of neutrophilic extracelluar traps (NETs)^57,58^. NETs are composed of decondensed chromatin that is released from the nuclear membrane and mixed with disarticulated histones and granules containing antimicrobial proteins before being ejected from the cell^55,58–60^. The expelled chromatin traps the offending microbes while simultaneously promoting destruction of the entrapped cells^55^. Interestingly, along with the granular proteins, disarticulated histones and short fragments of DNA (<100 bp) are also potent antimicrobials, likely increasing the antibiotic effect of NETs^59,61^.

Many bacteria have been shown *in vitro* to stimulate NETosis, including the oral bacteria *Porphyromonas gingivalis* and *Aggregatibacter actinomycetemcomitans*^62^. Moreover, certain bacteria subvert NETosis by producing extracellular nucleases (DNases) which are either bound to the cell membrane or secreted from the cell^63^. These nucleases free trapped bacteria by degrading the DNA backbone of the excreted NETs^55^. This activity is particularly prevalent during periodontal disease, and a wide range of oral bacteria including *P*. *gingivalis*, *Tannerella forsythia*, *Fusobacterium nucleatum*, and *Parvimonas micra* are able to produce extracellular nucleases^45^.

If host DNA is incorporated into dental calculus in an acellular form – either through NETosis or by another mechanism – the exposed DNA would be vulnerable to a variety of damaging processes, including both hydrolysis and extracellular nuclease activity. This may explain why, even within the same sample, human DNA within dental calculus exhibits a higher level of fragmentation than DNA derived from oral microbes^64^.

### Cell wall structure is not correlated with microbial DNA fragmentation or damage

It has been previously proposed that certain microbial cell wall attributes, such as the presence (Gram-positive) or absence (Gram-negative) of a thick peptidoglycan layer, may influence the preservation of microbial DNA and therefore contribute to biases in taxonomic analyses of archaeological dental calculus^13^. However, a subsequent investigation of four dental calculus samples failed to find such a correlation^26^. In this study, we test this hypothesis in 48 dental calculus samples and find no relationship between attributes such as cell wall peptidoglycan structure or the presence of an S-layer and DNA fragmentation or terminal cytosine damage patterns. This analysis does not preclude other aspects of cellular structure (e.g., spore formation or the presence of mycolic acids) that may impact aDNA preservation and were not tested in this study. Our analysis of reads assigned to specific bacteria suggests that fragmentation and damage patterns may be taxonomically structured. However, the consistency and biological basis of these patterns is beyond the scope of the data presented here.

### Loss of short AT-rich DNA fragments may contribute to taxonomic skew

Analysis of the relationship between genomic GC content, DNA fragment GC content, and DNA fragment length reveals an inverse relationship between DNA fragment length and GC content in taxa with low- and medium-GC genomes, suggesting a systematic loss of short AT-rich fragments. Short DNA fragments lack thermostability and are easily lost through denaturation. The melting temperature of short DNA fragments is primarily dependent on DNA sequence and length, in addition to environmental conditions and other factors^65^, and in general sequences with longer lengths and higher GC content have higher melting temperatures. Short DNA fragments from taxa with lower GC content genomes are expected to be more susceptible to loss through denaturation because their melting point for a given fragment length will be lower, and this may contribute to taxonomic skew. Consistent with this hypothesis, we found that high GC-content genera had slightly shorter median fragment lengths overall (Fig. 5b), which accords with a higher retention of short DNA fragments.

Although we observe these patterns in archaeological dental calculus, it is unclear if this is an artifact introduced during sequencing preparation or a naturally occurring taphonomic process. Importantly, this effect is weaker or absent from high genomic GC content genera (Fig. 6), which include many soil bacteria^66^. If the loss of short AT-rich fragments is primarily taphonomic and not methodological in nature, greater taxonomic skew may be expected in libraries generated using a single-stranded library preparation, which is known to retain a higher proportion of shorter DNA fragments than the double-stranded DNA library preparation method used in this study^67^. Documentation of potential taxonomic-specific biases in recovery of DNA is critical as they impact downstream interpretations of metagenomic data, affecting accurate description of these ancient microbial ecosystems.

In this study, we use metagenomic sequencing data to explore patterns of preservation in microbial- and host-derived DNA in a large and diverse set of paired archaeological dentin and dental calculus samples (n = 48 individuals, n = 96 samples). Our results confirm that dental calculus is a rich source of well-preserved oral microbiome DNA and a consistent source of highly fragmented and low abundance human DNA. We find that cell wall structure has no significant association with microbial DNA preservation, but that all samples exhibit systematic loss of short AT-rich DNA fragments, a trend that disproportionately affects taxa with low and moderate GC content genomes. Finally, we show that approximately one third of teeth retain DNA from the oral microbiome and thus dentin may serve as an alternative source of oral bacterial DNA in the absence of preserved dental calculus deposits. Our findings demonstrate that dental calculus provides a favorable environment for long-term DNA preservation.

## Methods

### Samples and DNA extraction

Paired dental calculus and dentin samples were obtained from seven geographically and temporally distinct sites: Middenbeemster in the Netherlands (n = 2, ca. 1611–1866 CE)^68^, Camino del Molino in southeastern Iberia (n = 2, cal. 2340–2920 BCE)^69^, Samdzong in Nepal (n = 2, cal. 400–650 CE)^70,71^, Arbulag Soum, Khövsgöl in Mongolia (n = 2, 930–1650 BCE)^72^, Anse à La Gourde in Guadeloupe (n = 2, cal. 975–1395 CE^26^), Norris Farms in IL, USA (n = 2, ca. 1300 CE)^17,73^, and Kilteasheen in Ireland (n = 36, ca. 600–1300 CE)^74^ (Supplementary Table S1). The first six sites were selected to represent global patterns of DNA preservation across diverse environments, burial contexts, and time periods. Remains and data from this site are referred to as the global dataset. A more extensive sampling of a single site in Kilteasheen, Ireland was performed to account for regional DNA preservation between individuals with similar burial contexts across a time transect of approximately six centuries. Remains and data from this site are referred to as the regional dataset.

### Global sample set

Samples were prepared for sequencing in a dedicated ancient DNA laboratory at the Laboratories of Molecular Anthropology and Microbiome Research (LMAMR) in Norman, Oklahoma, USA. With the exception of the previously processed Norris Farms samples (see Ozga *et al*.^17^), teeth were first decontaminated with bleach, then the calculus was separated using a dental scaler. The crown was separated from the root using a Dremel rotary tool. Partitioned tooth roots and calculus were further decontaminated via exposure to UV irradiation for 2 minutes. DNA extraction was performed as described by Ziesemer *et al*.^26^. Approximately 10–20 mg of dental calculus and 100 mg of dentin were crushed and then immersed in 1 ml of 0.5 EDTA for 15 minutes to remove any additional surface contaminants. Dental calculus samples were demineralized in a solution of 0.45 M EDTA and 10% proteinase K (Qiagen, the Netherlands) at 37–55 °C for 8–12 hours followed by room temperature incubation for five days until digestion was complete. Dentin samples were demineralized at room temperature. After 2 days, the EDTA supernatant was removed and refreshed with new EDTA and 50 μl of proteinase K (Qiagen, the Netherlands). Dentin samples were then left to demineralize for an additional 3 days at room temperature. The two dentin fractions were combined after digestion was complete. Prior to demineralization, all samples were centrifuged and the supernatant was used for DNA extraction using a phenol-chloroform-isoamyl alcohol (25:24:1) along with three blanks. Extracted DNA was isolated using a modified Qiagen MinElute silica spin column purification protocol. Extracted DNA was eluted twice for a total volume of 60 μl, and quantified using a Qubit High Sensitivity fluorometer (Life Technologies) (Supplementary Table S1).

### Regional sample set

Samples were prepared and extracted in the paleogenetics clean room at the Institute for Archaeological Sciences, University of Tübingen (INA). The surface of the dedicated sampling hood was cleaned with HPLC water and UV irradiated by an internal light source between uses. Any calculus was removed from the surfaces of the teeth using dental scalers that had been rinsed with bleach and HPLC water and UV irradiated for 10 minutes between uses. Large calculus samples were pulverized with a tube pestle. Teeth were then sectioned horizontally at the cementoenamel junction and dentin was drilled from the pulp chamber using a dental drill. For calculus samples weighing over 20 mg, half the pulverized material was carried over for extraction. For dentin samples over 70 mg, aliquots of approximately 50 mg were taken for extraction. Dentin and calculus samples were extracted using a previously described silica-based method^75^. In brief, samples were submerged in a digestion buffer with final concentrations of 0.45 M EDTA and 0.25 mg/mL proteinase K (Qiagen, the Netherlands) and rotated at 37 °C until decalcified. After incubation, samples were centrifuged and the supernatant was purified using a 5 M guanidine-hydrochloride binding buffer with High Pure Viral Nucleic Acid Large Volume kits (Roche, Switzerland). The extracts were eluted in 100 μl of a 10 mM tris-hydrochloride, 1 mM EDTA (pH 8.0), and 0.005% tween-20 buffer (TET). One extraction blank was prepared for every ten samples, and one positive control of cave bear bone powder was processed alongside each extraction batch to ensure efficiency. The extracts were quantified using a Qubit High Sensitivity fluorometer (Life Technologies) (Supplementary Table S1).

### Illumina library preparation

#### Global sample set

Approximately 100 ng of DNA was built into each Illumina shotgun library at the LMAMR, Norman, Oklahoma using NEBNext DNA Library Prep Master Set (E6076) and blunt-end modified Illumina adapters. Manufacturer’s instructions were followed with the exception of Nebulization. Blunt-end repair was performed using 50 μl reactions with 30 μl of DNA extract for each sample which was then incubated for 20 min at 12 °C and 15 min at 37 °C and purified using Qiagen MinElute silica spin columns following the manufacturer’s instructions. All samples were eluted in 30 μl. Following blunt-end repair, Illumina adapters were ligated in 50 μl reactions. Reactions were incubated for 15 minutes at 20 °C and purified using Qiagen QiaQuick columns before elution in 30 μl EB. Samples were then incubated for 20 minutes at 37 °C followed by 20 min at 80 °C in a final volume of 50 μl for adapter fill-in. Libraries were quantified using a real-time quantitative PCR assay (qPCR, Lightcycler 480 Roche) targeting the IS7 and IS8 sequences in the universal Illumina adapters (Supplementary Table S1). Libraries were amplified and dual-indexed in a 50 μl PCR reaction using 15 μl template, 25 μl of a 2 × KAPA U + master mix, 5.5 μl H_2_O, 1.5 μl DMSO, 1 μl BSA (20 mg/ml), and 1 μl of each forward and reverse index (10 μl μM). Thermocycling conditions were 5 min at 98 °C followed by 10–12 cycles of 15 seconds at 98 °C, 20 seconds at 60 °C, and 20 seconds at 72 °C, followed by a final elongation step for 1 minute at 72 °C. Amplified libraries were then purified using Agencourt AMPure XP beads and eluted in 30 μl EB. Samples were sent for sequencing on an Illumina HiSeq2500 using a paired-end, 200-cycle, rapid-run chemistry. Samples were sequenced to an average depth of 36 million reads (Supplementary Table S1), and blanks were sequenced to a depth of 0.4–1 million reads (Supplementary Table S10). Deeper sequencing of the blanks was not performed because a high degree of clonality was already achieved at this depth of sequencing.

#### Regional Sample Set

Double-stranded Illumina libraries were built using 10 μl of extract for each sample according to an established protocol^76^. Purification of the blunt-end repair and adapter ligation steps was performed using Qiagen MinElute columns. After the adapter fill-in step, the Bst polymerase was deactivated with a 20 minute incubation at 80 °C. A single library blank was used for every ten samples. The libraries were then quantified using real-time qPCR assay (Lightcycler 480 Roche) targeting the IS7 and IS8 sequences of the universal Illumina adapters (Suppelementary Table S1). Each library was assigned a unique pair of indices, added to the library over 2–15 indexing reactions per library. Libraries were double-indexed^77^ in 100 μl reactions using varied amounts of template and H_2_O based on library richness, 10 μl PfuTurbo buffer, 1 μl PfuTurbo (Agilent Technologies), 1 μl dNTP mix (25 mM), 1.5 μl BSA (10 mg/ml), and 2 μl of each indexing primer (10 μM). The reactions were purified, pooled, and eluted over MinElute columns in 50 μl TET. Efficiency of the indexing reactions was evaluated using a qPCR assay. Approximately one-third of each indexed library was amplified using 3–5 μl of template in 70 μl reactions with Herculase II Fusion DNA Polymerase (Agilent Technologies). Products for each sample were pooled and quantified using an Agilent Tape Station D1000 Screen Tape kit. Amplified sample and blank libraries were pooled into two 10 nM pools for shotgun sequencing. Samples and blanks were sequenced separately on Illumina NextSeq500 using single-end, 75-cycle, high-output kits. Samples were sequenced to a depth of approximately 5 million reads per library (Supplementary Table S1), and blanks were sequenced to a depth of 157,109 to 841,776 reads (Supplementary Table S10). Additionally, thirteen individuals of the regional sample set were re-sequenced using paired-end 150-cycle chemistry on an Illumina NextSeq500 so they could be included in fragment length analyses.

#### Computational Methods

Sequencing data from the global and regional sample sets were computationally processed identically using EAGER v1.92^78^. In brief, adapters were removed, paired-end data merged, and reads quality filtered using Clip&Merge^78^ with a minimum base quality of 20 and a minimum fragment length of 30. Processed reads were then taxonomically binned using MALT version 038^28^ and the full NCBI nucleotide database (‘nt’, April 2016) with an 85% identity threshold, a minSupportPercent threshold of 0.01, and a topPercent value of 1.0 (Supplementary Tables S2, S3, S14). Metagenomic profiles were analyzed with MEGAN6 CE^29^ and screened for specific taxonomic levels for fragment length and damage pattern profiles using a RMA file format parsing script^79^. Length and damage patterns were visualized using the ggplot2^80^ and lattice^81^ libraries in R. For all fragment length and damage rate analyses only those samples that were paired end sequenced were used. Additionally, only merged reads were considered to prevent artifacts brought on by unmerged pairs, the maximum length of which is fixed. Mapping to the human genome (hg19) was performed using BWA (−n 0.01, −l 1000, −q 30)^82^ as implemented in EAGER v1.92^78^. A normalized taxon table with species-level assigned reads (Supplementary Table S2) was exported from the bacterial and archaeal sub-trees in MEGAN and used to generate a Bray-Curtis taxonomic distance matrix in R using the vegan library (version 2.4–1)^83^. Principal coordinates were generated using the R ape library^84^ and visualized as a PCoA plot using ggplot2^80^. The same taxon table was used to produce microbial profiles of each sample according to isolation source within a nested classification scheme^85^. This scheme illustrates the relative environmental and human microbial contributions to each sample based on a species-by-species literature survey based on species name searches in PubMed. Pathobionts and opportunistic pathogens are designated as such when literature on the organism consistently presented it as a health threat, though it also may be a natural inhabitant of the human microbiome or soil. Assigned read counts for the classified species were tabulated and visualized using the Krona Excel Template^31^. Potential source contribution to samples was also calculated from summarized read quantities of the bacterial and archaeal sub-trees (Supplementary Table S3) using SourceTracker version 0.9.8^34^. Source accession numbers: ERR1017187, ERR1019366, ERR1022687, ERR1022692, ERR1034454, ERR1035437, ERR1035438, ERR1035441, ERR1039457, ERR1039458, ERR1043165, ERR1044071, ERR1044072, ERR1051325, SRR1631060, SRR1631061, SRR1631063, SRR1631064, SRR1633008, SRR3184100, SRR3184876, SRR3189411, SRR3189416, SRR3189418, SRS014107, SRS015650, SRS018665, SRS018975, SRS019029, SRS019129, SRS019387, SRS023538, SRS063215, SRS077312. The microbial classification scheme with Krona plots and SourceTracker were used in conjunction to control for potential biases related to the modern samples used in the latter and together present a layered representation of the types of microorganisms typically found in ancient dental calculus and dentin. Finally, GC content versus length profiles were generated using a custom python script (https://github.com/aemann01/gcLenCorPlots). When possible, analyses were run in parallel using GNU parallel^86^.

### Accession numbers

Genetic data have been deposited in the NCBI Short Read Archive (SRA) under the Bioproject accession PRJNA445215.

## Electronic supplementary material

Supplementary Information

Dataset 1
