# Supplementary material for: Differential preservation of endogenous human and microbial DNA in dental calculus and dentin
Source: Sci Rep. 2018 Jun 29;8:9822. doi: 10.1038/s41598-018-28091-9 (PMC6026117; doi:10.1038/s41598-018-28091-9)
Supplement: Supplementary file 1 — Supplementary Information [file 41598_2018_28091_MOESM1_ESM.pdf]

SUPPLEMENTARY INFORMATION

Differential preservation of endogenous human and microbial DNA in dental calculus and dentin

Allison E. Mann, Susanna Sabin, Kirsten Ziesemer, Åshild J. Vågene, Hannes Schroeder, Andrew T. Ozga, Krithivasan Sankaranarayanan, Courtney A. Hofman, James A. Fellows Yates, Domingo C. Salazar-Garcia, Bruno Frohlich, Mark Aldenderfer, Menno Hoogland, Christopher Read, George R. Milner, Anne C. Stone, Cecil M. Lewis, Jr., Johannes Krause, Corinne Hofman, Kirsten Bos, Christina Warinner

Correspondence to: warinner@shh.mpg.de

Supplementary Methods

Linear Discriminant Analysis (LDA) of Microbial Communities
MALT results for dentin, calculus, and blank samples (excluding NF47 and NF217 samples) were extracted as a taxon table representing the whole bacterial and archaeal sub-trees, with taxon abundances expressed as normalized, summarized read counts. Samples were classified by material type (dentin, n=45; calculus, n=47; blank, n=33). Any taxa that were not present in at least 30% of the samples in at least one class were excluded from the analysis. We conducted an LDA on the resulting taxon table with the LDA effect size (LEfSe) method, available on the Galaxy server (Segata et al. 2011). The alpha value for the Kruskal-Wallis test among classes was set to 0.05, and the minimum logarithmic LDA score threshold was set to 2.0.

Human Read Validation:
Reads mapped to the hg19 human reference genome using sensitive BWA (Li and Durbin 2009) mapping parameters (-n 0.01, -l 1000, -q 30) were de-replicated using DeDup as implemented in EAGER version 1.92 (Peltzer, Jäger et al. 2016). Next, de-replicated bam files were converted to fastq format using bedtools version 2.25.0-1 (bedtools bamtofastq) (Quinlan 2010) and where necessary split into forward, reverse, and merged reads. Three base pairs were trimmed from either side of all merged reads using fastx_trimmer from the FASTX-Toolkit version 0.0.14 (http://hannonlab.cshl.edu/fastx_toolkit/). Forward reads were trimmed of three base pairs exclusively on the 5' end of the read while reverse pair reads were trimmed for three base pairs on the 3' end. Trimmed reads were then remapped to the hg19 human reference genome using BWA with a higher mismatch penalty and quality mapping threshold so that approximately one mismatch would be allowed per 50 bases (-n 0.2, -l 1000, -q 37). The reads passing this quality threshold filter have a higher level of confidence of their proper assignment to the human genome. To further test the validity of these reads, they were then run through a lowest-common-ancestor algorithm via MALT version 038 using the full NCBI NT database with a percent identity threshold of 90%. Those reads that were assigned to the *Homo sapiens* node are recorded in Supplementary Table S9. Finally, to test whether these high confidence human reads are in fact ancient and not the result of background contamination, the original reads pre-damage trimming were pulled from the original fastq files, mapped to the hg19 human reference genome using BWA (-n 0.01, -l 1000, -q 30) in the EAGER v1.92 pipeline (Peltzer, Jäger et al. 2016) and assessed for terminal cytosine deamination patterns using mapDamage version 2.0 (Jonsson, Ginolhac et al. 2013). For all calculus samples and most dentin samples, the percent endogenous and damage patterns both pre- and post-strict map filtering are comparable, confirming the observed pattern of a low but consistent human aDNA retrieval from calculus and a variable yield from dentin (Supplementary Figure S5).

As an additional assessment of exogenous contamination, mitochondrial contamination estimates were generated using Schmutzi for all samples demonstrating sufficient (i.e. at least five-fold) coverage (Renaud et al. 2015) of the human mitochondrial reference genome (Supplementary Table S10).

Bacterial fragment analyses:
Reads that mapped to the species node and all higher resolution taxonomic nodes were extracted from the MALT results for all bacteria of interest. To limit the impact of erroneous mapping only those reads with damage at the terminus of the read were considered. For all fragment length analyses, only merged reads were analyzed.

Supplementary Figures

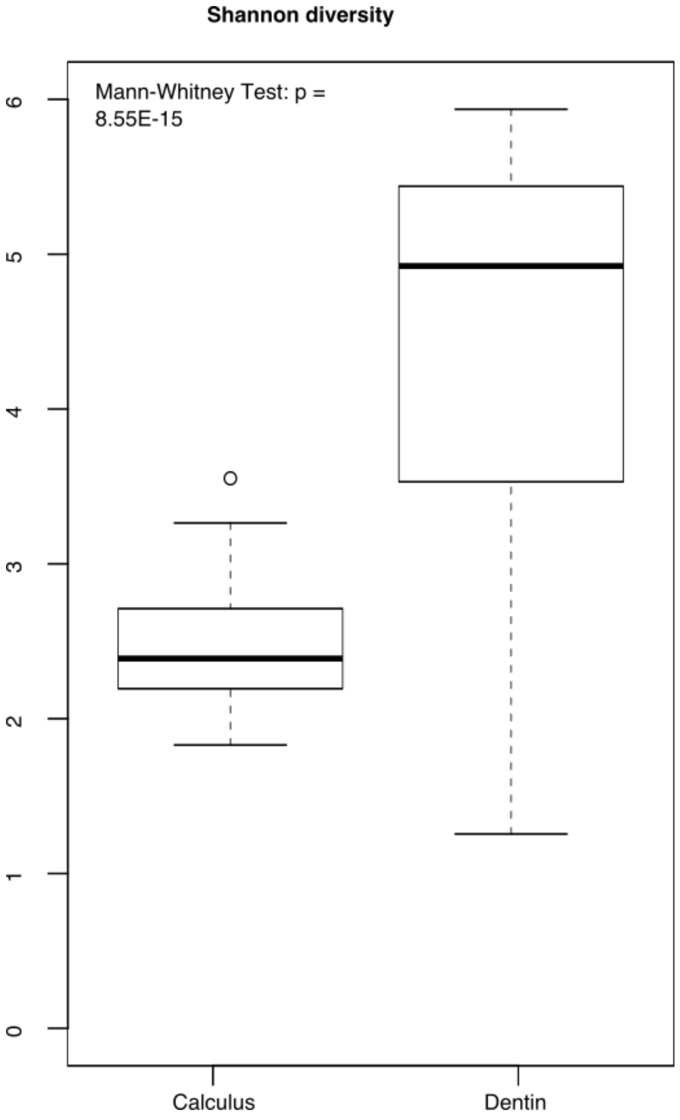

Supplementary Figure S1: Boxplots of alpha diversity (Shannon Weaver Index) for all dentin and dental calculus samples. Diversity among dental calculus is significantly lower (Mann-Whitney-U p = 8.55E-15) as compared to that found in the dentin samples.

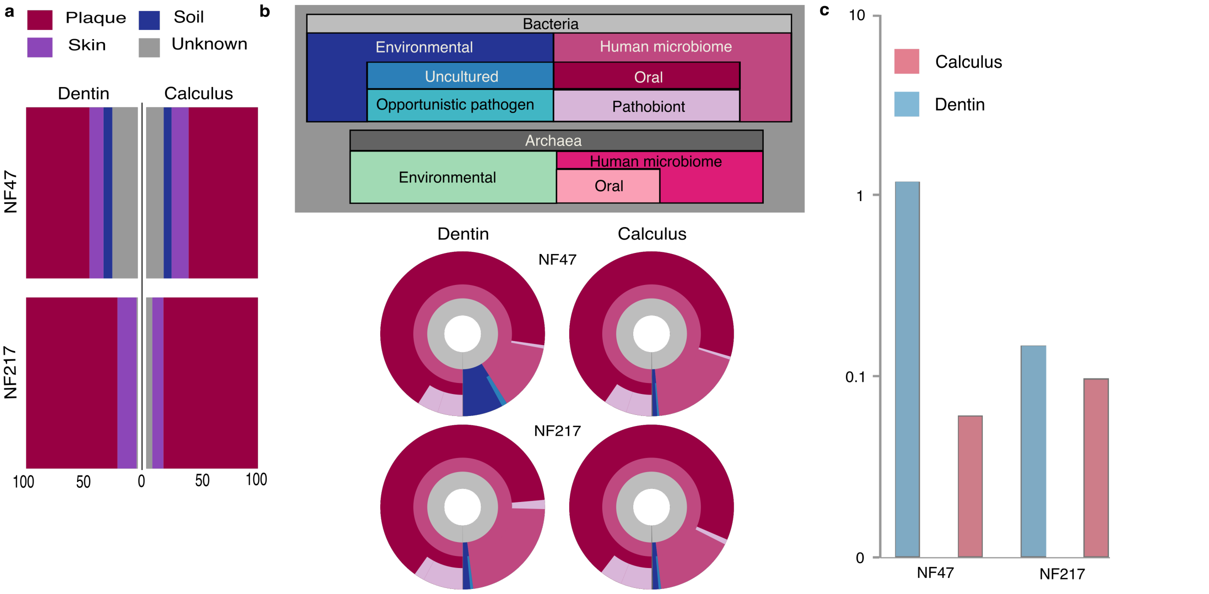

Supplementary Figure S2: Likely signal of carious lesions on two dentin samples. (**a**) Bar chart displaying Bayesian SourceTracker results of the estimated genetic contributions from human oral and environmental microbial sources. (**b**) The donut plots are constructed from nested classification of species-level MALT results. The microbial communities in the Norris Farms dentin and calculus samples have a high oral contribution, as demonstrated by both the SourceTracker bar plots and the MALT classification donut plots. **(c)** Log transformed ($\log_{10}$) proportion human endogenous DNA in NF47 and NF217 dentin and calculus samples reflecting a relatively high proportion of microbial DNA in the Norris Farms dentin samples.

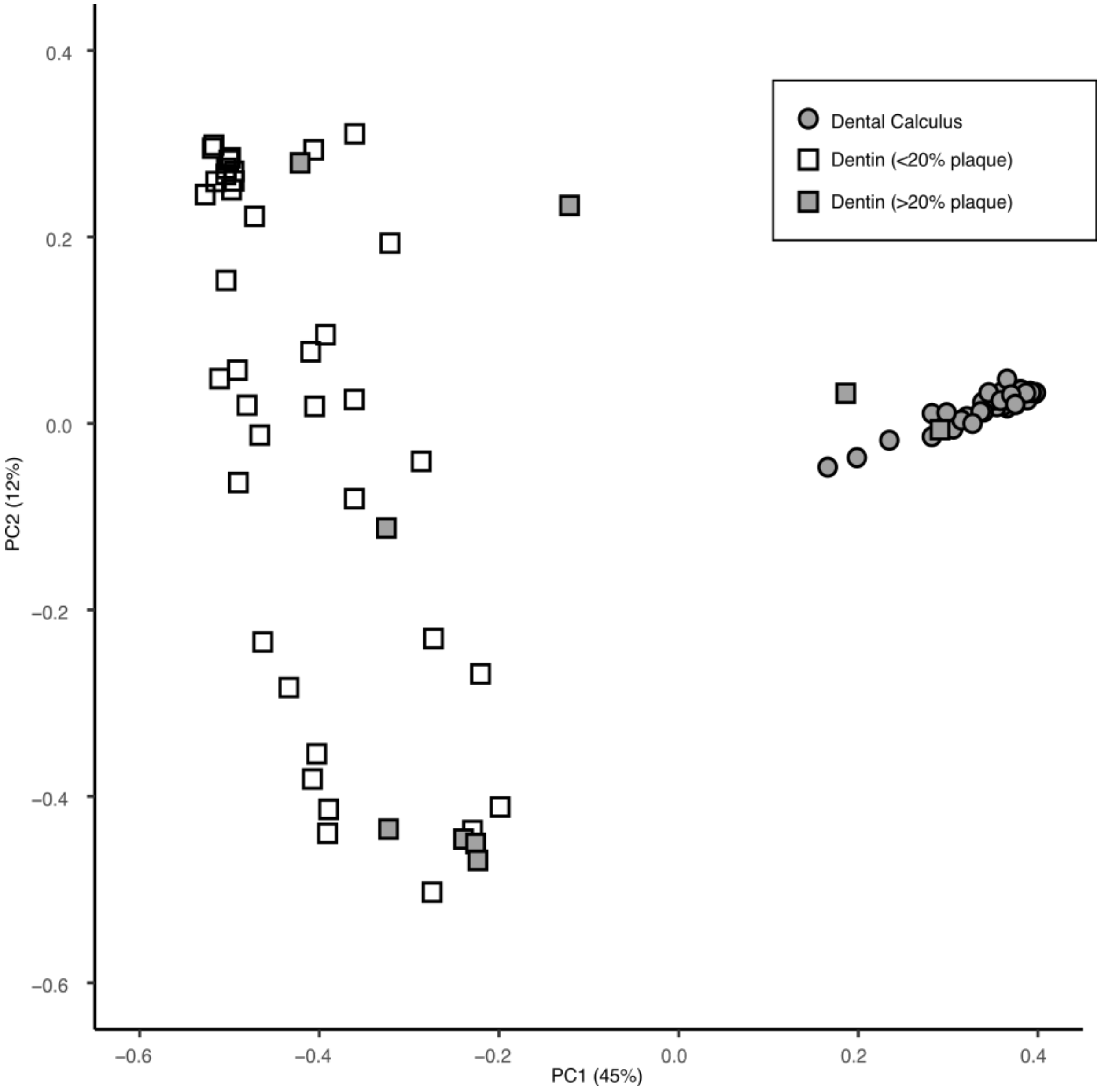

Supplementary Figure S3. Principal Coordinates Analysis (PCoA) of Bray-Curtis distances of all bacterial and archaeal species-level assignments from dental calculus and dentin. Dental calculus is represented as circles and dentin is represented as squares. Color indicates estimated contribution of oral taxa**.** Black symbols are those samples that have a predicted proportion of oral contribution of 20% or more, illustrating that some dentin samples have an unexpectedly high oral signature, though most do not cluster with the dental calculus samples, indicative of a non-biological community.

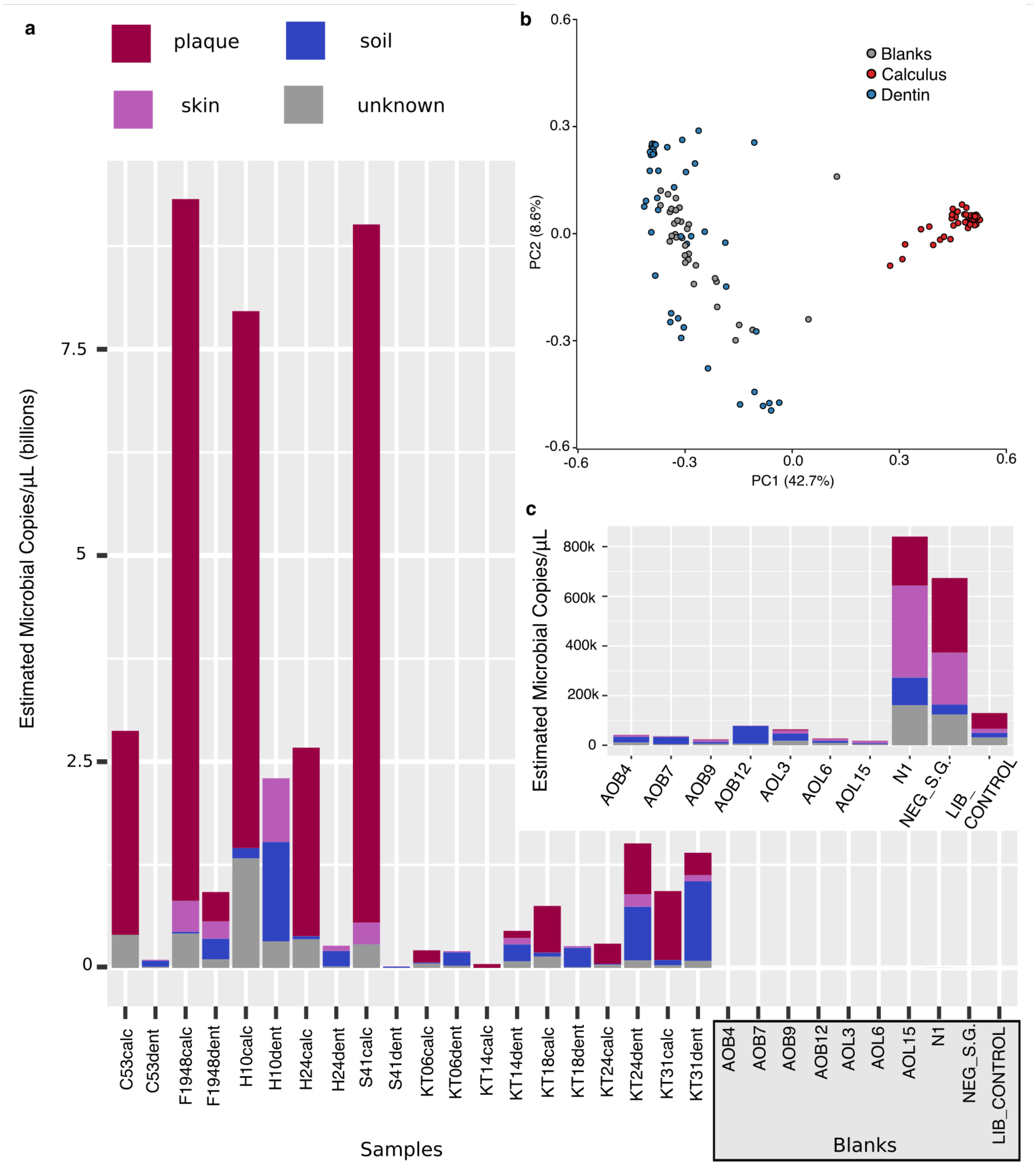

Supplementary Figure S4. Comparison of microbial DNA quantity and composition between samples and blanks. (a) Estimated number of copies of microbial DNA per microliter of pre-indexed library, with proportion of predicted sources indicated. Predicted sources of microbial DNA were calculated for each sample using SourceTracker. Total microbial copies/µL was calculated for each sample by subtracting the proportion of endogenous human DNA (Supplementary Tables S9 and S10) from the total copies/µL (Supplementary Table S1). **(b)** PCoA of Bray-Curtis dissimilarity generated from a normalized taxon table of bacterial and archaeal species-level abundances for all samples (excluding NF47 and NF217) and blanks. **(c)** Enhanced view of the estimated source proportions and number of copies of microbial DNA per microliter of pre-indexed blank libraries; enhanced view is shown because the quantity of DNA measured in the blanks was too low to be visible in panel **(a).**

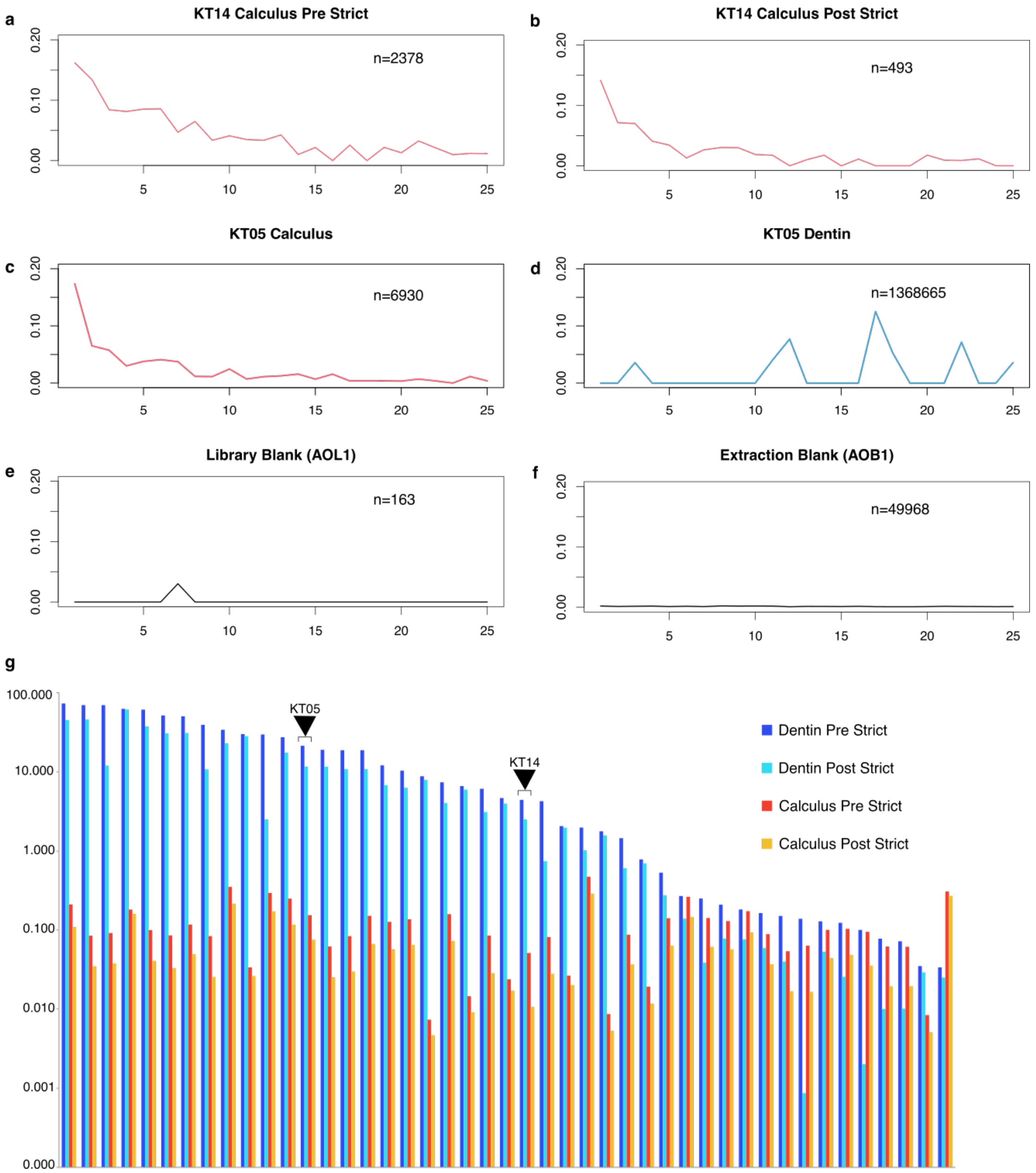

Supplementary Figure S5. Validation of ancient human DNA authenticity. (a) Terminal cytosine deamination rates of reads mapped to the human genome from a single calculus sample (KT14) before extra human validation steps. **(b)** Damage rate of human reads from calculus sample KT14 after human validation. While the damage rate of human reads post-validation drops, a clear damage signal, consistent with authentic ancient DNA is still observed. **(c-d)** Termnial cytosine deamination rates of reads mapped to the human genome from a calculus and dentin pair where the calculus exhibits a clear damage signal consistent with authentic ancient DNA while the dentin sample is consistent with modern contamination. **(e-f)** Terminal cytosine deamination of a library blank and extraction blank. Neither blanks have an observed damage signal, consistent with modern DNA. (**e**) Proportion human endogenous content for all paired samples both before and after strict mapping (see Supplementary Methods). While in all cases the proportion of human endogenous content drops after strict mapping, the effect is minor for most samples. Major drops are detected in certain dentin samples.

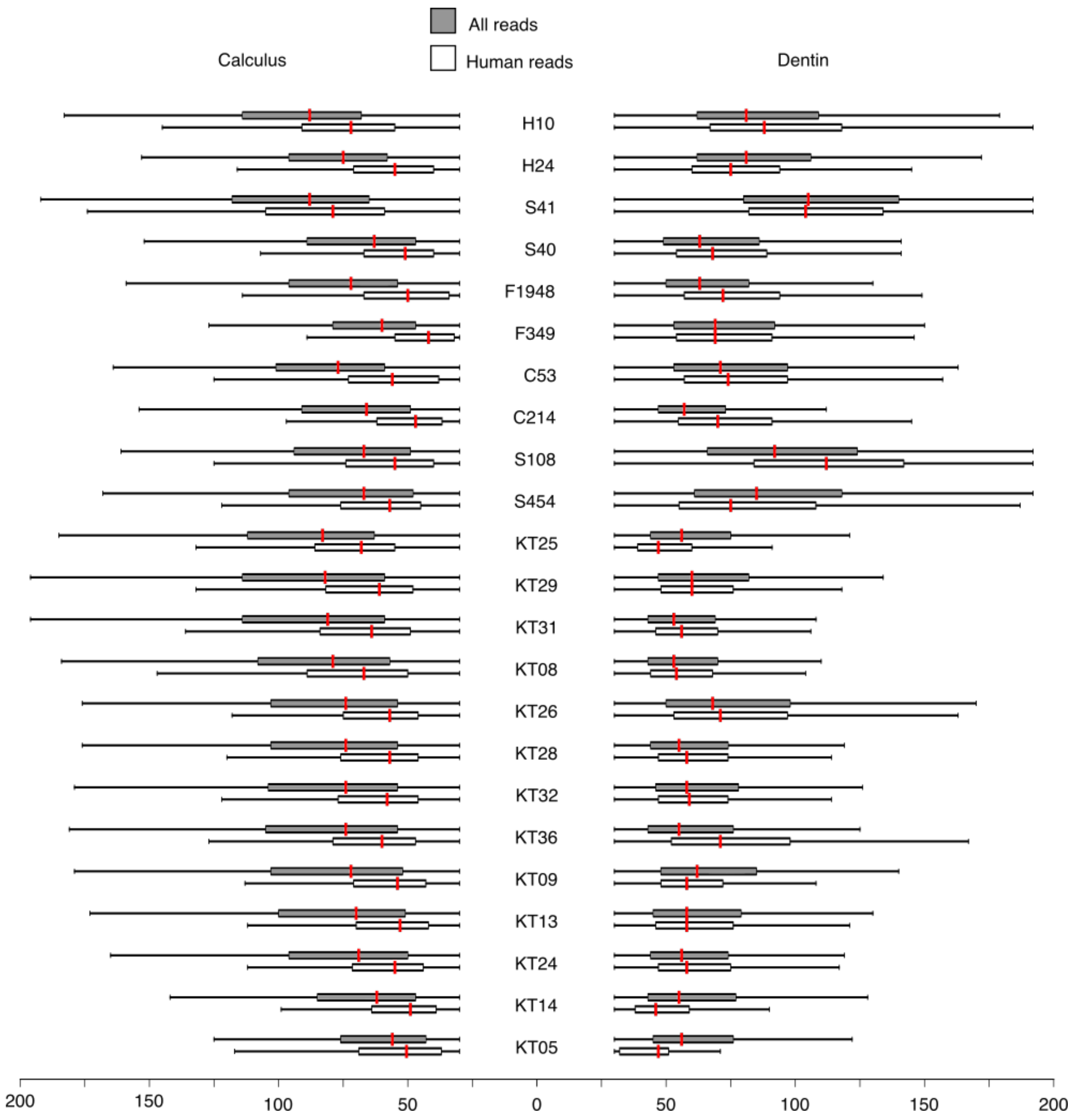

Supplementary Figure S6: Boxplots comparing fragment size distributions of human and non-human reads in each sample. For calculus, human reads are consistently shorter than those that map to microbial sources. By contrast, no consistent pattern is observed for dentin samples.

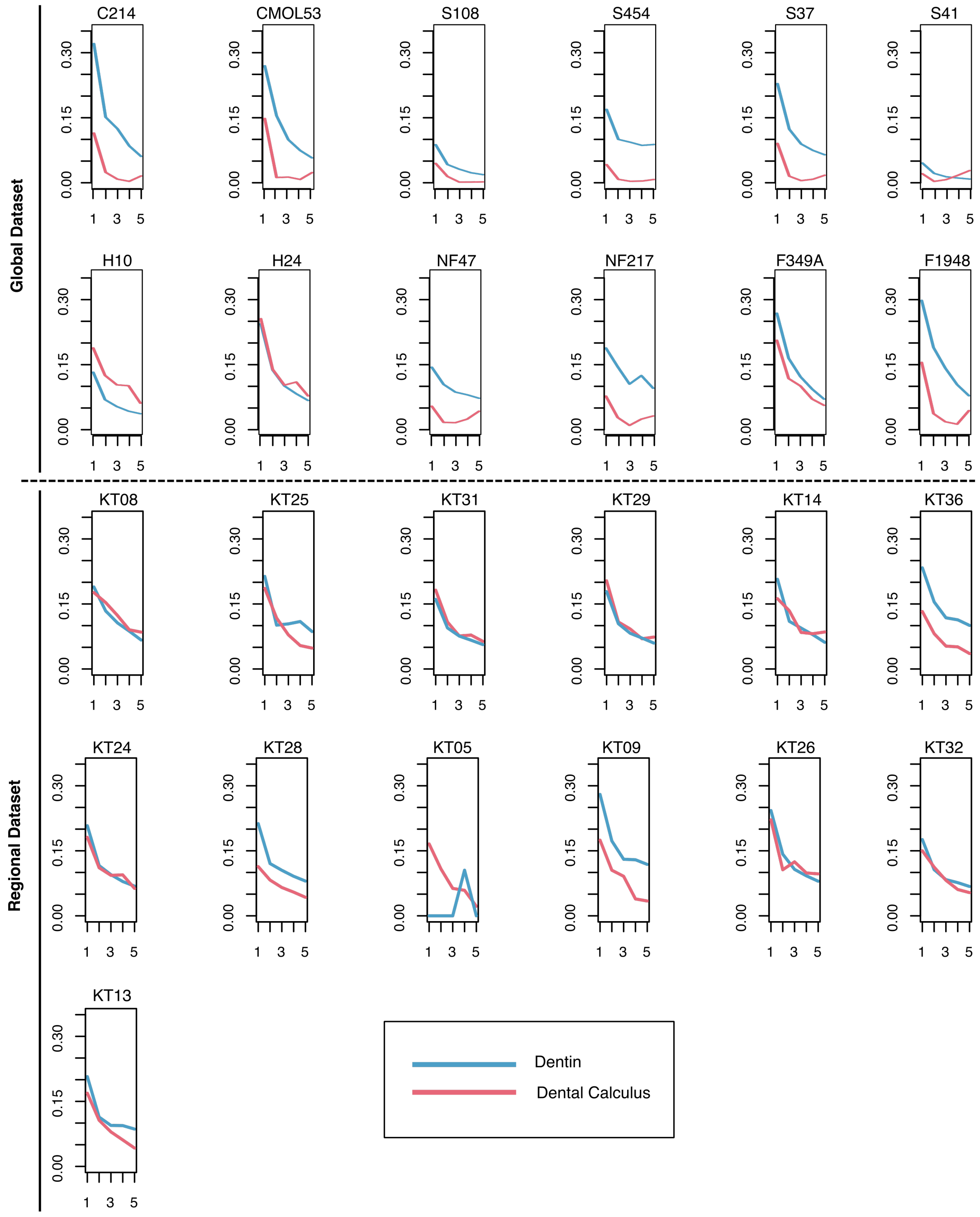

Supplementary Figure S7. Differences in microbial damage patterns among paired dentin and dental calculus is sample-specific. Cytosine damage patterns for a subset of paired dentin (blue) and dental calculus (red) samples. While most dental calculus samples have a lower initial deamination rate than their dentin pair in the global dataset, this pattern is not consistently observed in the regional dataset, possibly the result of differences in laboratory preparation.

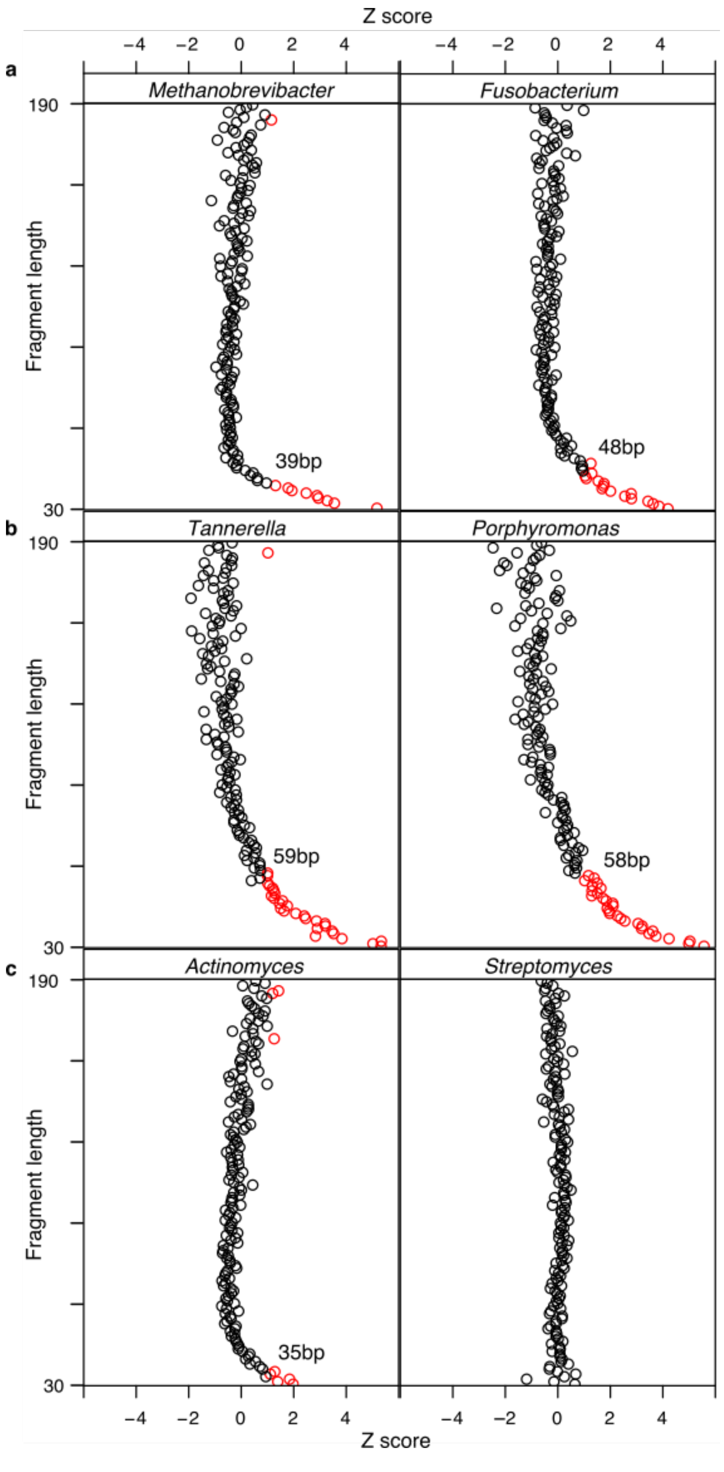

Supplementary Figure S8. Fragment length deviation from expected mean GC content for selected bacterial genera. Bacterial genera are organized by expected genomic GC content wherein (a) are low genomic GC taxa, (b) are moderate genomic GC taxa and, (c) are high genomic GC taxa. Each point represents a single length bin's mean deviation from the overall mean of all reads mapped to the genus. Red points are those length bins that are one or more Z scores deviated from the mean GC content. For each genus the read length bin at which a major deviation can be seen (±1 z score) is noted on the graph. For low or medium genomic GC content genera, this length threshold occurs at a higher fragment length than those with high genomic GC content.

Supplementary References

Jonsson, H., A. Ginolhac, M. Schubert, P. L. Johnson and L. Orlando (2013). "mapDamage2.0: fast approximate Bayesian estimates of ancient DNA damage parameters." Bioinformatics **29**(13): 1682-1684.

Li, H. and R. Durbin (2009). "Fast and accurate short read alignment with Burrows-Wheeler Transform." Bioinformatics **25**: 1754-1760.

Peltzer, A., G. Jäger, A. Herbig, A. Seitz, C. Kniep, J. Krause and K. Nieselt (2016). "EAGER: efficient ancient genome reconstruction." Genome Biology **17**(1): 60.

Quinlan, A. (2010). "BEDTools: a flexible suite of utilities for comparing genomic features." Bioinformatics **26**(6): 841-842.

Renaud, Gabriel et al. (2015). "Schmutzi: estimation of contamination and endogenous mitochondrial consensus callling for ancient DNA." Genome Biology **16**: 224

Segata, N., et al. (2011). "Metagenomic biomarker discovery and explanation." Genome Biology **12(6)**: R60
